# The Impact of Cytokines on Health-Related Quality of Life in Adolescents with Allergic Rhinitis

**DOI:** 10.3390/biomedicines12020428

**Published:** 2024-02-13

**Authors:** Ljiljana Krsmanović, Nenad Arsović, Dejan Bokonjić, Vladimir Nešić, Zoran Dudvarski, Dragana Pavlović, Milena Dubravac Tanasković, Siniša Ristić, Nikolina Elez-Burnjaković, Radmila Balaban, Branislava Ćurčić, Radenko Ivanović, Nikolina Vuković, Maja Vuković, Marija Milić, Bojan Joksimović

**Affiliations:** 1 University Hospital Foča, 73300 Foča, Bosnia and Herzegovina; ljiljana.krsmanovic@ues.rs.ba (L.K.); dbokonjic@gmail.com (D.B.); draganatosovic@gmail.com (D.P.); branislavacurcic@gmail.com (B.Ć.); radenko84ivanovic@yahoo.com (R.I.); nklngljnn@gmail.com (N.V.); 2 Faculty of Medicine Foča, University of East Sarajevo, 73300 Foča, Bosnia and Herzegovina; arsovic9@gmail.com (N.A.); dudvarski1809@gmail.com (Z.D.); menadub@gmail.com (M.D.T.); risticsinisa@yahoo.com (S.R.); nikolinaa85@hotmail.com (N.E.-B.); radmilabalaban@yahoo.com (R.B.); majavukovic81@gmail.com (M.V.); 3 Clinic of Otorhinolaryngology and Maxillofacial Surgery, Clinical Center of Serbia, 11000 Belgrade, Serbia; vlanesic@gmail.com; 4 Faculty of Medicine Belgrade, University of Belgrade, 11000 Belgrade, Serbia; 5 Department of Epidemiology, Faculty of Medicine, University of Pristina Temporarily Seated in Kosovska Mitrovica, 38220 Kosovska Mitrovica, Serbia; marija.milic@med.pr.ac.rs

**Keywords:** allergic rhinitis, cytokines, health-related quality of life, adolescents

## Abstract

Background: Frequent episodes of nasal symptoms are the usual clinical manifestations (CM) of allergic rhinitis (AR) and have a significant negative impact on health-related quality of life (HRQoL) in adolescents. The purpose of this cross-sectional study was to test the hypothesis that cytokines in nasal mucus may be associated with HRQoL in adolescents with AR. Methods: European Quality of Life 5 Dimensions 3 Level Version (EQ-5D-3L), “The Adolescent Rhinoconjunctivitis Quality of Life Questionnaire” (AdolRQLQ) and the Total 4 Symptom Score (T4SS) scoring system were administered to 113 adolescents with AR, nonallergic rhinitis (NAR) and to healthy control subjects. Nasal secretions were sampled and tested for 13 cytokines using a multiplex flow cytometric bead assay. Results: The AR group had significantly lower EQ-5D-3L (0.661 ± 0.267 vs. 0.943 ± 0.088; *p* < 0.001) and higher AdolRQLQ total scores (2.76 ± 1.01 vs. 1.02 ± 0.10; *p* < 0.001) compared to the control group. The AR group had higher concentrations of IL-1β (*p* = 0.002), IL-6 (*p* = 0.031), IL-8 (*p* < 0.001), IL17-A (*p* = 0.013) and IL-18 (*p* = 0.014) compared to the control group, and IL-1β, IL-6, IL17-A and IL-18 were significantly (*p* < 0.050) increased with disease progression. Cytokines IL-1β, IL-6, as well as severe CM, were identified as significant predictors of lower HRQoL in adolescents with AR. Conclusions: This study identified IL-1β, IL-6, as well as severe CM, as predictors of lower HRQoL in adolescents with AR. However, these results should only serve as a starting point for additional confirmation research.

## 1. Introduction

Rhinitis may be classified into two groups: those with a concurrent allergy (allergic rhinitis, AR) and those without an allergy but who still suffer from nasal symptoms (nonallergic rhinitis, NAR) and without specific immunoglobulin E (IgE)-mediated hypersensitivity. One of the most common non-communicable chronic diseases globally, AR is caused by IgE, which is generated by plasma cells in response to allergen exposure. It is a long-term IgE-mediated Th2 inflammatory disease of the nasal mucosa that involves several different cells and cytokines, caused by a type I hypersensitivity reaction in sensitized people when they are exposed to common inhalation allergens [1]. According to epidemiological studies, the incidence of AR ranges from 20–40%, with some nations reporting rates as high as 50% [2], affecting over 500 million individuals in the world across all age groups, particularly the pediatric population [3]. Prevalence of AR is estimated to be 15.1% to 37.8% in the adolescent population [4], which is particularly different from children and adults, because it is characterized as a developmental period from 12 to 17 years of age, marked with many physical, psychological and social changes as the adolescents move beyond parental control [5]. AR can be divided into two categories: seasonal (SAR) and perennial (PAR), based on the kind of allergen and the disease pattern. Pollens and other outdoor allergens are the primary cause of SAR, whereas indoor allergens like dust mites, cockroaches, and pets can cause PAR [6]. Due to shared systemic inflammatory processes, AR frequently coexists with other illnesses, including atopic dermatitis (AD), rhinosinusitis, rhinoconjunctivitis, and asthma in particular. This coexisting aspect is commonly referred to as “the atopic March” [7,8]. While the prevalence of AR as a comorbidity in asthmatic patients is considerably higher, ranging from 70% to 90%, 40% to 50% of people with AR also have asthma [9]. Exposure to allergens can lead to hyperreactivity of various cells, which leads to recruitment of eosinophils, activation of mast cells, dendritic cells (DCs), epidermal keratinocytes, lymphocytes and goblet cells in various parts of the airways. These activated immune cells initiate a cascade of local and systemic inflammatory reactions [10]. It is hypothesized that many of the signs and symptoms of AR are caused by uncontrolled inflammation [11]. Cytokines produced by the above-mentioned immune cells participate in the development of hypersensitive disease, and subtypes of T helper-Th cells (Th1 and Th2) play an important role in the development and progression of AR. These immune cells produce a number of cytokines, which have been found to cause a number of pro-inflammatory reactions, but also reactions that have the opposite anti-inflammatory effects. The outcome depends on the balance of these cytokines and biomolecules [10]. It has been known that the AR is related to enhancement of Th2 lymphocyte responses, as evidenced by elevated levels of Th2-profile cytokines in the nasal mucosa, including interleukin (IL)-4, IL-5 and IL-13 [12], which plays a key role in the pathogenesis of AR. In addition to the initial Th2/Th1 concept, other cytokines such as Th17 cell cytokines (IL-17, IL-22 and IL-21), IL-3, granulocyte/macrophage colony-stimulating factor (GM-CSF), and tumor necrosis factor α (TNF-α) have been discovered to be significantly involved in the pathogenesis of AR [10]. In addition, localized infiltration of eosinophils, which is linked to a suppression of interferon-γ (IFN-γ) production, promotes the inflammatory process. These modified responses are responsible for the disease symptoms [12].

Frequent episodes of nasal symptoms, such as a runny nose, sneezing, pruritus, and nasal congestion, are the usual manifestation of AR and have a significant negative impact on health-related quality of life (HRQoL) [13]. AR has an impact on patients’ HRQoL in a variety of ways, such as sleep quality, academic and professional performance, heightened levels of fatigue, melancholy, an elevated risk of motor vehicle accidents, and modified physical and social functioning [14]. This can cause patients to feel depressed, irate, frustrated and to withdraw socially [15]. More than 80% of individuals with moderate to severe AR are thought to have had difficulties with their everyday activities, compared to 40% of people with mild AR, which leads to lower productivity at school or at work [16]. Due to the rising need for therapy and medical care, this presents a financial burden on the community [17,18]. 

Understanding the burden of AR in populations of adolescents is crucial because of the distinct developmental profile of teenagers in comparison to younger children and adults. This is because understanding this population’s development may have implications for the optimal disease management [5]. It has been proven that persistent immune dysfunction leads to upregulation of proinflammatory cytokines, such as interleukin IL-1β, IL-6 and TNF-α, which have been associated with fatigue, social withdrawal, irritability and depressive moods [19]. These behavioral problems can consequently affect HRQoL in patients with AR [20].

Nevertheless, no study has yet investigated the impact of inflammatory cytokines on HRQoL in adolescents. Given that the current disease activity tools are not well suited to capturing the underlying immunological activity of AR in adolescents and that studies assessing the impact of cytokines on HRQoL in this population are lacking, the purpose of this cross-sectional study was to test the hypothesis that cytokines in nasal mucus may be associated with HRQoL and the severity of clinical manifestations (CM) in adolescents with AR.

## 2. Materials and Methods

### 2.1. Study Design and Data Collection

This is a cross-sectional study of an adolescent population (*n* = 113, mean age 14.39, 54.1% girls) in the territory of the municipality of Foča, Republic of Srpska, Bosnia and Herzegovina. All subjects that participated in the study and their guardians were informed of the purpose and procedure of the examination, and written consent was obtained from both participants and guardians. The research was conducted from 1 October to 31 December 2022, while laboratory analyses were carried out from 1 February to 1 May 2023. The research was conducted in accordance with the Helsinki Declaration and the principles of Good Clinical Practice. The diagnosis of AR was based on the Allergic Rhinitis and its Impact on Asthma (ARIA) guidelines [2]. All participants from the AR group met the inclusion criteria of AR diagnosis, such as a positive result from a skin-prick test (SPT) and/or a specific IgE (>0.350 IU/mL), and symptoms typical of AR for >3 years. A positive result of the SPT was verified as an induration on the skin greater than 3 mm in diameter compared to the negative control. SPT was performed on all subjects outside the pollen season for standard inhalation allergens in the Dermatology-Allergology Clinic, and specific IgE analyses were performed in the Laboratory for Biochemistry of the University Hospital in Foča. We excluded those with other inflammatory or immunologic diseases, a history of immunotherapy, and systemic steroid/antiallergy medication consumption within the four weeks prior to enrollment. The diagnosis of NAR was established in children with symptoms and signs of AR and negative SPT (the list of tested allergens is presented in Table 1). The adolescents were also divided by the presence of comorbidities. Study participants were divided into three groups. The first group included children with AR, the second group consisted of subjects with NAR, and the third (control) group included healthy children (subjects without symptoms and signs of AR, negative SPT and with negative data in the family history of atopic diseases).

### 2.2. Instruments

Epidemiological data were collected during the visit to physicians through questionnaires that the adolescents filled out. The socio-demographic questionnaire (age, gender, information about possible comorbidities), a general questionnaire for determining the quality of life, the “European Quality of Life 5 Dimensions 3 Level Version” (EQ-5D-3L), a specific questionnaire for assessing the quality of life in children with AR, “The Adolescent Rhinoconjunctivitis Quality of Life Questionnaire” (AdolRQLQ), and the Total 4 Symptom Score (T4SS) for assessing severity of clinical manifestations were used. 

EQ-5D-3L consists of the EQ-5D-5L descriptive system and the EQ visual analog scale (EQ-VAS). The EQ5D-5L description system consists of five levels (no problems, slight problems, moderate problems, severe problems, and extreme problems) corresponding to five dimensions (mobility, self-care, usual activities, pain/discomfort, and anxiety/depression). The EQ-VAS measures the participant’s self-rated health (“How good is your health TODAY?”) on a vertically numbered visual analogue scale from 0 to 100. “The worst health you can imagine” at 0 and “The best health you can imagine” at 100 are its endpoint labels. One level from each of the five dimensions is combined to create the EQ-5D-5L health states, which result in a total of 3125 potential health states. For instance, “11111” denotes no issues in any of the five dimensions, whereas “12345” indicates no issues with mobility, mild issues with self-care (such as bathing or clothing), moderate issues with daily tasks, severe pain or discomfort, and severe anxiety or depression [21,22]. In this work, the EQ-5D-5L total score (index) was computed using a Korean valuation set [23]. 

Rhinoconjunctivitis-specific HRQoL was assessed with the validated AdolRQLQ. AdolRQLQ is employed for participants aged 12–17 years. The questionnaire is composed of 25 questions and 6 domains (practical problems, symptoms unrelated to AR, nasal symptoms, ocular symptoms, activity limitation and emotional/mental problems) [24,25]. A seven-point Likert scale (0–6) is used to indicate each response, ranging from “not troubled/bothered at all” to “extremely troubled/bothered”. The mean total score and the mean score for the domains were separately calculated [25,26].

Anamnestic data on AR symptoms (based on a subjective scoring system for evaluating the severity of four symptoms of AR patients: nasal congestion, sneezing, runny nose and nasal itching) were assessed in adolescents diagnosed with AR using a questionnaire Total 4 Symptom Score (T4SS) [27].

### 2.3. Cytokine Analyses

From all subjects, nasal secretions were sampled using the absorption technique. All samples from subjects with AR were taken during the period of existing symptoms. The wooden sticks with cotton wool on top were held in the nasal cavity behind the junction of the skin and mucous membrane for 60 s. The sampled nasal secretion swabs were placed in an Eppendorf tube, volume 2 mL, which had 1 mL transfer medium (phosphate-buffered saline with 50 µg/mL gentamicin, 340 U/mL penicillin G and 500 µg/mL fungizone) for 30 min, due to the diffusion of cytokines into the transfer medium, and then it was kept at a temperature of 4 °C for a maximum of 2 h. Nasal secretions were centrifuged at 1000 rpm for 10 min to separate cellular components. After centrifugation, the supernatant was stored and frozen at −70 °C until cytokine detection [28,29]. Concentrations of thirteen cytokines (IL-1β, IFN-α2, IFN-γ, TNF-α, MCP-1 (CCL2), IL-6, IL-8 (CXCL8), IL-10, IL-12p70, IL-17A, IL-18, IL-23 and IL-33) were measured. Cytokine concentrations were determined by using multiplex fluorescent beads labeled with anti-cytokine antibodies (Biolegend, San Diego, CA, USA) on the flow cytometer (Attune Acoustic Focusing Cytometer, Applied Biosystems, Themo Fisher, Waltham, MA, USA), according to the manufacturer’s instructions [30,31,32] in the Center for Biomedical Science, Faculty of Medicine Foča, University of East Sarajevo.

### 2.4. Data Analysis

The methods of descriptive and analytical statistics were used in the paper. Among the methods of descriptive statistics, measures of central tendency and measures of variability were used, namely: arithmetic mean with standard deviation (SD) and relative numbers for categorical variables. Among the methods of analytical statistics, the Mann–Whitney test was used for bound samples and the Kruskal–Wallis test, with pairwise post hoc testing, was used to determine differences between three groups. Of the nonparametric tests, the chi-square test was used to assess the difference between the groups. The correlation was conducted with the help of Spearman’s correlation coefficient ®. The usual value of *p* < 0.05 was taken as the level of statistical significance of differences. Linear regression analysis was used to examine the predictive power of the variables that showed statistical significance in the univariate analysis on the outcome variables of interest (HRQoL variables). Results were statistically analyzed in SPSS software package version 21.0 (Statistical Package for Social Sciences SPSS 21.0 Inc., IBM, New York, NY, USA).

## 3. Results

The research included 133 adolescents, of which 72 (54.1%) were female. The mean age of the subjects was 14.39 ± 1.68 years. The group with AR consisted of 55 (41.4%) subjects, the group with NAR consisted of 34 (25.6%) subjects, while the control group consisted of 44 (33.1%) subjects. No significant difference was observed between the groups of respondents in relation to their gender and age. Out of the total number of respondents, 63 (47.4%) had sinusitis, 6 (4.5%) secretory otitis, 4 (3%) OSA, 2 (1.5%) had polyps, 8 (6%) asthma, 25 (18.8%) atopic dermatitis, and 31 (23.3%) had conjunctivitis. Out of the total number of respondents, 44 (33.1%) had no CM, 23 (17.3%) had a mild form of CM, 34 (25.6%) had a moderate form, while 32 (24.1%) had a severe form of CM. A high statistically significant difference (*p* < 0.001) was observed between the groups of subjects in relation to the degree of severity of CM. All subjects from the control group were free of CM; however, the moderate (40%) and severe forms of CM (43.6%) were significantly more frequent in the group of subjects with AR compared to the group with NAR (35.3% vs. 23.5%). Subjects with AR had a significantly more frequent overall occurrence of comorbidities compared to subjects from the group with NAR (83.6% vs. 70.6%) (*p* < 0.001). Also, sinusitis (*p* < 0.001), asthma (*p* = 0.002), AD (*p* < 0.001) and conjunctivitis were significantly more frequent in the group of respondents with AR compared to the group with NAR (76.4%, 14.5%, 32.7%, 45.5% vs. 61.8%, 0%, 20.6% and 17.6%). Out of a total of 133 subjects, 55 subjects, who made up the group with AR, had a positive result on the SPT of standard allergens. Thirty-four (61.8) had a positive test for mites, 21 (38.2) for birch tree pollen, 22 (40) for hazelnut pollen, 29 (52.7) was allergic to grass pollen, 30 (54.5%) for Cat’s tail grass pollen, 21 (38.2%) was positive for rye pollen. Thirteen (23.6%) subjects were allergic to ambrosia, 5 (9.1%) were allergic to wild wormwood, 2 (3.6%) to the fungus *alternaria*, 5 (9.1%) to the brown cockroach, 3 (5.5%) to cat and 6 (10.9%) to dog hair. The adolescents from AR group were divided based on the type of AR on SAR (*n* = 35; 63.6%) and PAR (*n* = 21; 36.4%). Twenty-one adolescents (38.2%) were mono-sensitized, while 34 (61.8%) were poly-sensitized (Table 1).

**Figure 1:**
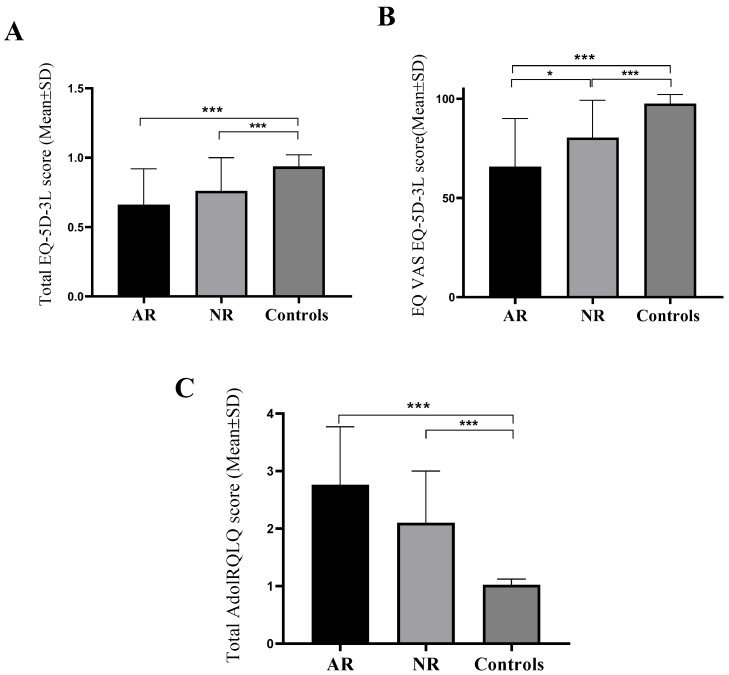
Mean values of the total index score of the EQ-5D-3L questionnaire (**A**), EQ-VAS score (**B**) and total AdolRQLQ score (**C**) between groups of respondents. AR—allergic rhinitis, NAR—nonallergic rhinitis, EQ-5D-3L—European Quality of Life 5 Dimensions 3 Level Version, AdolRQLQ—the Adolescent Rhinoconjunctivitis Quality of Life Questionnaire, Mean ± SD (standard deviation), Kruskal–Wallis test, with pairwise post hoc testing between groups, * *p* < 0.050; *** *p* < 0.001.

Figure 1A shows that the group of subjects with AR had significantly (*p* < 0.001) lower EQ-5D-3L total index score values (0.661 ± 0.267) compared to the control group of subjects (0.943 ± 0.088). Also, subjects from the group with NAR (0.763 ± 0.243) had significantly (*p* < 0.001) lower values of the total EQ-5D-3L index score compared to the control group. No difference in EQ-5D-3L scores between the group with AR and the group with NAR was observed (Figure 1A). The group of subjects with AR possessed significantly (*p* = 0.016) lower EQ-VAS values (65.72 ± 24.29) compared to the group of subjects with NAR (80.44 ± 18.76) and the control group of subjects (97.56 ± 4.49) (*p* < 0.001). Also, subjects from the group with NAR had significantly (*p* < 0.001) lower EQ-VAS values compared to the control group (Figure 1B). The group of subjects with AR had significantly (*p* < 0.001) higher values of total AdolRQLQ score (2.76 ± 1.01) in comparison to the control group of subjects (1.02 ± 0.10). Also, respondents from the group with NAR (2.10 ± 0.90) had significantly (*p* < 0.001) higher values of the total AdolRQLQ score compared to the control group. No difference in values between the group with AR and the group with NAR was noticed (Figure 1C).

Subjects with AR (23.6%) had a significantly more frequent occurrence of moderate mobility problems in comparison to subjects from the group with NAR (8.8%) and the control group of subjects (0%) (*p* = 0.001). Moderate problems in usual activities were significantly (*p* < 0.001) more frequent in the group of subjects with AR (52.7%) compared to the group with NAR (26.5%) and the control group (0%). No difference in the frequency of problems in the EQ-5D-3L domain of self-care between the groups was observed. Subjects with AR (52.7%) had a significantly more frequent occurrence of moderate problems with pain and discomfort compared to subjects from the group with NAR (44.1%) and the control group of subjects (11.4%) (*p* < 0.001). Moderate problems in the EQ-5D-3L domain of anxiety and depression were significantly (*p* < 0.001) more frequent in the group of subjects with AR (49.1%) compared to the group with NAR (38.2%) and the control group of subjects (18.2%).

The group of respondents with AR had significantly (*p* < 0.001) higher values (worse quality) in the domain of practical problems of the AdolRQLQ (3.05 ± 1.15) in relation to the control group of subjects (1.03 ± 0.14). Also, respondents from the group with AR held significantly (*p* = 0.012) lower values in the domain of practical problems compared to the group with NAR (2.38 ± 1.45). The difference was also noticed between the group of subjects with NAR and the control group of subjects (*p* = 0.040). Subjects with AR (2.65 ± 1.19) (*p* < 0.001) and subjects with NAR (2.23 ± 1.17) (*p* < 0.001) had significantly higher values (worse quality) in the domain of symptoms not related to allergic sneezing in comparison to the control group of subjects (1.03 ± 0.14). No difference in the values of this domain between the groups of subjects with AR and NAR was observed. The group of subjects with AR (3.72 ± 1.41) (*p* < 0.001) and subjects with NAR (2.88 ± 1.46) (*p* < 0.001) possessed significantly higher values (worse quality) in the nasal symptoms domain compared to the control group of subjects (1.03 ± 0.16). No difference in the nasal symptom domain values between the groups of subjects with AR and NAR was noted. Subjects with AR (2.38 ± 1.42) (*p* < 0.001) and subjects with NAR (1.59 ± 0.94) (*p* = 0.023) had significantly higher values (worse quality) in the eye symptom domain compared to the control group of subjects (1.00 ± 0.00). Also, a difference was observed between the group of subjects with AR and the group with NAR (*p* = 0.008). The values of the activity domain of the AdolRQLQ questionnaire were significantly higher (worse quality) in the group of subjects with AR (2.24 ± 1.46) (*p* < 0.001) in compared to the control group of subjects (1.00 ± 0.00). No difference in activity domain values was noticed between the groups of subjects with AR and NAR. Subjects with AR (2.34 ± 1.22) (*p* < 0.001) and subjects with NAR (1.78 ± 0.99) (*p* < 0.001) had significantly higher values (worse quality) in the domain of emotional symptoms compared to the control group of subjects (1.04 ± 0.21). Also, a difference was observed between the group of subjects with AR and the group with NAR (*p* = 0.032) (Table 2).

**Figure 2:**
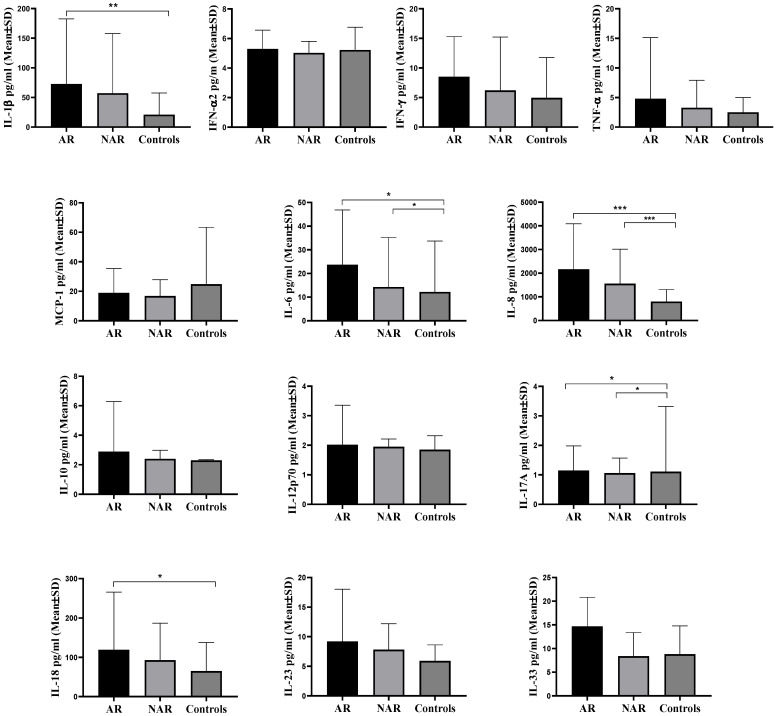
Mean values of IL-1β, IFN-α2, IFN-γ, TNF-α, MCP-1 (CCL2), IL-6, IL-8 (CXCL8), IL-10, IL-12p70, IL-17A, IL-18, IL-23 and IL-33 in nasal mucosa of subjects of different groups. AR—allergic rhinitis, NAR—nonallergic rhinitis, IFN–interferon, TNF-α—tumor necrosis factor alpha, MCP-1—Monocyte Chemotactic Protein-1, Mean ± SD (standard deviation), Kruskal–Wallis test, with pairwise post hoc testing between groups, * *p* < 0.050, ** *p* < 0.010, *** *p* < 0.001.

Subjects with AR had significantly higher (*p* = 0.002) IL-1β values (72.70 ± 109.82 pg/mL) compared to subjects from the control group (21.01 ± 36.54 pg/mL), while no difference between the group with NAR and the control group of subjects was observed. Subjects from the group with AR (23.73 ± 23.12 pg/mL) (*p* = 0.031) and the group with NAR (14.24 ± 20.97 pg/mL) (*p* = 0.044) possessed significantly higher IL-6 concentration values in comparison to the control group of subjects (12.20 ± 21.51 pg/mL). No difference in IL-6 levels was noticed between the group with AR and NAR. Subjects from the AR group (1829.73 ± 1833.15 pg/mL) (*p* < 0.001) and the group with NAR (1555.17 ± 1460.60 pg/mL) (*p* < 0.001) held significantly higher values of IL-8 compared to the control group of subjects (803.43 ± 502.07 pg/mL). No difference in IL-8 values between the groups of AR and NAR was observed. Subjects from the AR group (1.15 ± 0.83 pg/mL) (*p* = 0.013) and NAR group (1.06 ± 0.51 pg/mL) (*p* = 0.016) had significantly higher values of IL-17A compared to the control group of subjects (1.11 ± 2.21 pg/mL). No difference in IL-17A values between the group with AR and NAR was noted. Subjects from the AR group possessed significantly higher (*p* = 0.014) IL-18 values (119.12 ± 146.71 pg/mL) in comparison to the control group of subjects (64.98 ± 72.31 pg/mL). No difference in IL-18 values between the group with AR and NAR was observed, nor between the groups with NAR and the control group of subjects. There were no differences in the mean values of cytokines IFN-α2, IFN-γ, TNF-α, MCP-1 (CCL2), IL-10, IL-12p70, IL-23 and IL-33 in nasal mucosa of subjects between different groups (Figure 2).

Table 3 shows the mean values of inflammatory cytokines (IL-1β, IFN-2α, IFN-γ, TNF-α, MCP-1, IL-6, IL-8, IL-10, IL-12p70, IL-17A, IL-18, IL-23 and IL-3) between groups of subjects with allergic rhinitis divided according to the severity of CM. No significant difference was observed in the values of IFN-2α, IFN-γ, TNF-α, MCP-1, IL-8, IL-10, IL-12p70, IL-18, IL-23 and IL-33. However, it was observed that the concentration of IL-1β (*p* = 0.012) in nasal mucus was significantly higher in subjects with AR who had a severe form of CM compared to subjects with a mild form of CM (72.77 ± 117.84 vs. 40.90 ± 43.41pg/mL). IL-6 was also present in significantly (*p* = 0.028) higher concentrations in adolescents with severe CM when compared to the group with a mild form of the disease (48.99 ± 92.91 vs. 14.68 ± 23.09 pg/mL). The concentration of IL-17A was significantly (*p* = 0.021) higher in subjects with AR who had a severe form of CM compared to subjects with a mild form of CM (4.50 ± 0.50 pg/mL vs. 0.80 ± 1.60 pg/mL). Also, IL-18 levels were significantly (*p* = 0.018) increased in a group with severe CM when compared to the group with mild CM of AR (142.46 ± 170.93 pg/mL vs. 42.61 ± 0.17 pg/mL) (Table 3).

Examining the correlation between the concentration of inflammatory cytokines and the total EQ-5D-3L total score, only a weak negative correlation (r = −0.293; *p* = 0.030) was observed with IL-17A. Subjects who had a worse HRQoL measured with the EQ-5D-3L questionnaire had significantly higher concentrations of IL-17A. No significant association was noted between other inflammatory cytokines and total index EQ-5D-3L scores. The correlation between the total score of the AdolRQLQ and IL-12p70 was weakly positive (r = 0.299; *p* = 0.027). Subjects who had a worse quality of life (higher values of the total AdolRQLQ score) had significantly higher concentrations of IL-12p70. No significant association was observed between inflammatory cytokines and the total EQ-VAS score (Table 4).

Table 5 shows the multivariate regression analysis of the cytokine profile as a predictor of the quality of life (EQ-5D-3L and AdolRQLQ total scores) in models controlled for severity of clinical manifestations in adolescents with AR. In the “EQ-5D-3L model”, the variables that showed significance in previous univariate analysis were multivariately analyzed. The variables that, by multivariate regression analysis, showed predictive significance for the development of a lower health-related quality of life measured by the EQ-5D-3L questionnaire in our subjects were higher concentrations of IL-1β (*p* = 0.008), IL-6 (*p* = 0.043) and severe clinical manifestations (*p* = 0.037). IL-1β (β = 0.400) compared to IL-6 (β = 0.201) and clinical manifestations (β = 0.137) had the highest standardized coefficient values, so it can be considered that this cytokine is the best predictor of a lower health-related quality of life in a model controlled for clinical manifestations in adolescents with allergic rhinitis. However, in a “AdolRQLQ model” controlled for severity of clinical manifestations, only IL-1β (β = 0.389; *p* = 0.009) and severe clinical manifestations (β = 0.230; *p* = 0.037) were identified as significant predictive factors associated with a lower health-related quality of life, where IL-1β was the best predictor of a lower adolescent rhinoconjunctivitis health-related quality of life (Table 5).

## 4. Discussion

The aim of this study was to evaluate the impact of inflammatory cytokines on HRQoL in adolescents with AR and to identify differences in cytokine levels in relation to severity of clinical manifestations. This study showed that IL-1β and IL-6 were associated with lower HRQoL in adolescents with AR. Our study revealed that out of 131 adolescents, 55 (41.4%) had AR and one fourth had NAR (25.6%). The AR group of respondents had a significantly more frequent appearance of severe forms of clinical manifestations and overall occurrence of comorbidities, such as sinusitis, asthma, AD and conjunctivitis when compared to the NAR and control croup of adolescents. A study by Molgaard et al. [33] which examined differences between AR and NAR in a sample of 1186 showed that the prevalence of AR is even higher (77%) when compared with our study and confirmed that adolescents with AR had significantly frequent comorbidities such as asthma (*p* = 0.006), sinusitis (*p* = 0.006) and food allergies (*p* = 0.004) [33]. SPT and/or serum IgE findings were previously used as main criteria for diagnosing AR or NAR. However, a recent review article from Mortada and Kurowski [34] showed that this diagnosis can be achieved with more certainty if nasal allergen provocation tests (NAPT) are used. Many patients with a subtype of AR, local allergic rhinitis (LAR), were misdiagnosed as having NAR, which consequently deprived them of specific allergy treatment. It was proven that LAR can be highly suspected when the following clinical signs and symptoms are present: typical clinical symptoms (sneezing, rhinorrhea, and nose itching), absence of serum allergen-specific IgE, negative SPT, and a positive NAPT. Because in our study we did not use NAPT, which is one of the limitations of our study, it is possible that some NAR cases were in fact LAR cases, in which systemic IgE sensitization cannot be determined either through SPT or by measuring allergen-specific IgE in serum [35]. 

Inadequate management of AR can result in a number of previously mentioned comorbid conditions, all of which can worsen HRQoL [36,37]. Many studies have shown that both AR and its comorbidities are frequently followed by significantly lower HRQoL of children, adolescents and adults [38,39,40,41]. A study by Hillerich et al. [38] proved that in patients suffering from AR, the level of comorbidities was a statistically significant factor associated with low general HRQoL measured by the EQ-5D-3L questionnaire. Adolescents with AR experience a range of issues that could lower HRQoL. In addition to practical issues that also appear in adults, such as a need to rub the nose and eyes, blow the nose repeatedly, carry tissues, and take medication, adolescents could face several other constraints [42]. For example, adolescents could struggle in school because of learning impairment or be unable to engage in activities with friends and family, including playing sports on the grass or going camping, which are likely to trigger allergic reactions. Furthermore, adolescents who are unable to properly integrate with their peers may have emotional disturbances and feelings of isolation, which can result in frustration, despair, and rage [43]. These results highlight how important it is to properly diagnose and treat AR in order to prevent additional impairment of HRQoL by slowing down the onset and minimizing the severity of comorbidities. In our study, we found a significantly worse general (EQ-5D-3L total and domains scores) and specific adolescent rhinoconjunctivitis HRQoL (AdolRQLQ total and domains scores) in adolescents with AR when compared to adolescents with NAR and the control group. Our participants with AR were significantly restricted in mobility and usual daily activities and had problems in terms of pain/discomfort and anxiety/depression more often when compared to adolescents with NAR. Furthermore, the AdolRQLQ questionnaire showed that practical problems, nasal and ocular symptoms, and emotional/mental problems were significantly higher in a group with AR when compared to the NAR and control groups. A real-life prospective Spanish study examined HRQoL among adolescents (aged 12 to 17 years) with AR using the same instrument (AdolRQLQ) [44]. Before beginning the trial, adolescents had to be treated for at least two months with intranasal corticosteroids and oral antihistamines. When comparing treatment responders to adolescents who self-categorized as nonresponders to pharmacologic treatment, AdolRQLQ scores were considerably worse in the latter group. Stronger nasal symptoms, including obstruction (r = 0.64), anterior rhinorrhea (r = 0.59), itching (r = 0.55), sneezing (r = 0.56), and hyposmia (r = 0.54), were shown to be correlated with worse total AdolRQLQ scores in linear regression analysis [44]. 

AR is an inflammatory allergic disorder characterized by the development of nasal mucosal inflammation followed by the production of numerous cytokines by activated immune cells, including T cells, eosinophils, macrophages and mast cells. Additionally, allergic inflammation is linked with a shift in the ratio of Th1 to Th2 cell production of cytokines towards a Th2 predominance [45]. Due to natural allergen exposure, Th2 lymphocytes proliferate with the release of cytokines such as IL-1, IL-3, IL-4, IL-5, IL-6, IL-9, IL-10, IL-13, IL-17A, IL-18 and GM-CSF, and these inflammatory mediators play important roles in the pathogenesis of AR [46,47,48]. In the present study, the flow cytometry-based immunofluorescent assay kit was used to detect the levels of human cytokines in nasal mucosa IL-1β, IFN-α2, IFN-γ, TNF-α, MCP-1 (CCL2), IL-6, IL-8 (CXCL8), IL-10, IL-12p70, IL-17A, IL-18, IL-23 and IL-33 in adolescents with AR with different severity of the disease. We found that adolescents with AR had significantly higher concentrations of proinflammatory cytokines IL-1β, IL-6, IL-8, IL17-A and IL-18 when compared to the control group of adolescents, while values of IL-6, IL-8 and IL-17A were significantly higher in the NAR group when compared to the control group. Likewise, as AR worsened, IL-1β, IL-6, IL17-A and IL-18 levels gradually increased, suggesting that these four cytokines have a significant correlation with the progression of AR.

IL-6 is predominantly a proinflammatory cytokine which is released in the late phase of the allergic response [49], while Th2 IL-10 is an anti-inflammatory cytokine whose main role is to suppress the release of pro-inflammatory cytokines [50]. Ciprandi et al. [51] discovered that when other inflammatory variables are coordinated, IL-6 can trigger the release of particular allergy mediators and exacerbate AR. Numerous studies using samples of nasal mucus indicate that AR is associated with an increase in IL-4 levels [52,53,54]. Conversely, the production of IFN-γ is decreased in the nasal mucus of AR patients [55], which is confirmed by our results, where we have shown that the concentration of IFN-γ in nasal mucus was not different between the AR, NAR and control groups. The literature does, however, provide conflicting information about cytokine levels of IL-6 and IL-10 in AR [56], in adolescents with AR and asthma [57]. Some authors did not identify any difference of IL-6 concentrations between AR patients and healthy individuals; however, in many studies the concentrations of IL-1β and TNF-α were significantly higher in AR patients [56,57]. Scavuzzo et al. [46] showed that patients with AR showed higher levels of IL-4 (*p* < 0.001) and IL-10 (*p* < 0.05) and lower levels of IL-6 (*p* < 0.05) in the nasal mucus compared to healthy control subjects. This result could be explained by the dual effect of IL-4, because this cytokine suppresses the synthesis of IL-6 by having pro-inflammatory properties in the early stages of the disease [58] and the fact that Scavuzzo et al. [46] did not categorize their patients by the stage of AR. However, most studies found a significant increase in IL-6 [47,53] and no difference in IL-10 between the AR and control groups [59,60], which is consistent with our findings. Our finding that IL-6 and IL-17A were significantly higher in AR than in the NAR and control groups, and that both cytokines significantly increased with disease progression, is confirmed in a recent study by Gao et al. [47]. Similarly, many studies declared this finding [61,62,63] and confirmed that IL-17A levels are related to clinical severity in AR [62]. IL-17A, as an important member of the IL-17 family, can induce the activation of T lymphocytes, regulate signaling pathways (P38 mitogen-activated protein kinase) and stimulate the production of inflammatory cytokines, such as IL-6 and IL-8, thus starting a chain reaction of inflammatory airway responses [64]. A study by Gao et al. [47] even proved that IL-6 and IL-17A could be used as biochemical indicators for the diagnosis of AR in its early stages, that a high concentration of these cytokines was a significant risk factor for inducing AR, and that the CM of AR can be reduced and the disease can be controlled by inhibiting the levels of IL-6 and IL-17A. The production of eosinophilic chemokines and the infiltration of eosinophilic granulocytes can both be inhibited by elevated levels of IL-17A, which enhances the inflammatory response [65]. Furthermore, compared to healthy controls, AR patients’ peripheral blood and nasal mucosa had higher levels of IL-17A-positive cells, according to other studies [66,67]. Th17 cells appear to have a role in the neutrophil infiltration process that takes place during the acute phase of an allergic reaction [68]. Additionally, IL-17 activates Th2 cells that are specific to allergens, resulting in the synthesis of serum IgE and an accumulation of eosinophils, which suggests a regulatory function in the Th2-allergic immune response [68].

Consistent with previous reports [69,70,71,72,73], our study found significantly elevated IL-1β and IL-18 in participants with AR vs. the control group, and both were increased in the severe form of AR when compared to the group with a mild form of CM. Previous studies have shown increased IL-1β in the nasal mucus of patients with AR and that it acts as a key factor in the promotion of occurrence and development of AR [71,72,73] via the ROS-NLRP3-Caspase1-IL-1β signaling pathway [74,75]. It has even been proposed that IL-1β may be a potential biomarker of AR [76]. IL-1β is immediately released from cells within 1 to 2 h after allergen exposure [77] and gradually returns to the basal concentrations after 24 h [78]. The expression of IL-1β varies in different stages of AR, with the expression being significantly higher in the early and late stages, according to Bachert et al. [32], and its release may be related to the activation of T lymphocytes and endothelial cells. In terms of structure and pro-inflammatory qualities, IL-18—formerly known as the IFN-c-inducing factor—is a pro-inflammatory cytokine that is closely connected to the IL-1 family. Both IL-18 and IL-1β are generated as inactive precursors that require cleavage by the IL-1β converting enzyme (ICE or caspase-1) to become active proteins. These two cytokines share an analogous signaling route [77,79]. We found that IL-8 was also significantly increased in the AR group of participants when compared to the NAR and control groups. The proinflammatory cytokine IL-8 is important in eosinophil chemotaxis, and many studies concluded that patients with AR had a substantial increase in IL-8 release in response to allergen exposure [69,70]. A study by Verhaeghe et al. [80] demonstrated that IL-1β and IL-18 were significantly up-regulated in nasal secretions in AR and that IL-18 production was associated with persistent inflammatory processes. Even though our results did not show significant increases in IL-33 in adolescents with AR when compared to the NAR and control groups, the levels of IL-33 in AR group were higher in comparison to the NAR and control groups. IL-33, which is an alarmin, is localized in the nucleus of human endothelial and epithelial cells [81], and it is released in response to allergens but also to non-allergic stimuli, so it can be increased in nasal secretions in patients with AR and NAR [82]. A study by Baumann et al. [83] showed significantly higher concentrations of IL-33 in AR patients when compared to the control group and an inverse correlation (*r* = −0.61, *p* = 0.02) of nasal levels of IL-33 with disease severity. This finding complements our finding of IL-33 levels in nasal secretions being negatively correlated with severity of disease. However, even though the concentration of IL-33 was higher in adolescents with AR and its values decreased with the severity of the disease, these changes were not statistically significant in our study. 

Many studies concluded that AR had a negative effect on rhinoconjunctivitis [84,85,86] and general HRQoL [87,88,89] of adolescents, which is in line with our findings. It is commonly known that exposure to allergens activates mast cells, which in turn release mediators as well as cytokines that can attract and activate neutrophils, eosinophils, and Th2 lymphocytes, among other inflammatory cells. Common nose symptoms in AR are brought on by these inflammatory processes [90] and the intensity of these responses is associated with the severity of the disease [11,91]. However, studies in the literature that examine the impact of the cytokine profile on HRQoL of adolescents with AR are rare. This is the first time that association of cytokines measured in nasal mucosa with HRQoL in adolescents with AR is presented in a research study. When a correlation analysis was made between total EQ-5D-3L, EQ-VAS and AdolRQLQ scores and cytokine levels in the AR group, a negative correlation was detected only between IL-17A and total EQ-5D-3L score, and a positive correlation between IL-12p70 and total AdolRQLQ score, which means that higher levels of these two proinflammatory cytokines correlate with lower EQ-5D-3L and AdolRQLQ quality of life of subjects with AR. However, regression analysis showed that only IL-1β, IL-6 and the severity of CM were identified as significant predictive factors associated with lower HRQoL measured by EQ-5D-3L, and IL-1β and severe CM were associated with lower HRQoL measured by AdolRQLQ. IL-1β, IL-6 and IL-17A are proinflammatory cytokines, which can explain their increase in the severe forms of AR and the role of IL-1β and IL-6 as predictors of lower HRQoL in subjects with AR. One of the first studies that examined the relationship between HRQoL and immunological parameters was by Ciprandi et al. [91]. These authors found a significant association between eosinophil count in nasal mucosa and HRQoL [91], which was the first evidence that lower HRQoL in AR is strictly associated with allergic inflammation. A recent study by Sheha et al. [92] examined the correlation of serum IL-17A with disease severity and HRQoL of patients with AR. The authors demonstrated that patients with AR had significantly (*p* < 0.001) higher serum levels of IL-17A when compared to healthy controls and that IL-17A positively correlated with the visual analog scale (VAS) score (r = 0.302; *p* = 0.011), while the correlation between IL-17A and AdolRQLQ was negative but not significant [92]. The only study that investigated the correlation between nasal mucus cytokine levels and HRQoL in AR was in 76 adult patients undergoing endoscopic sinus surgery for chronic rhinosinusitis [11]. The authors found that IL-4 correlated significantly with the rhinologic domain (r = 0.250, *p* = 0.030) of the 22-item Sino–Nasal Outcome Test (SNOT-22) and IL-6 correlated with the 8-item Short Form Health Survey (SF-8) total score (r = 0.350, *p* = 0.020), general health (Rs = 0.340, *p* = 0.030), and emotional (r = 0.470, *p* = 0.002) scores [11].

We are aware that one of the limitations of the present study is the small sample size of the study population. Our study’s shortcomings also include its monocentricity, the potential for incomplete or erroneous data from patient medical files, non-use of NAPT to differentiate LAR cases from NAR cases, and the research population’s heterogeneity, which can be a major source of bias. Also, we did not measure concentrations of the main Th2 cytokines IL-4, IL-5 and IL-13, which can be a shortcoming because of their important role in the pathogenesis of AR and potential to affect the severity of clinical manifestations. A large-scale multicenter study should be performed to further elucidate the importance of measuring inflammatory cytokines and HRQoL in AR patients.

## 5. Conclusions

In conclusion, cytokines IL-1β, IL-6, IL-8, IL17-A and IL-18 play important roles in AR and their values are significantly higher in AR participants from healthy subjects. The concentrations of IL-1β, IL-6, IL17-A and IL-18 increase with the disease progression. Furthermore, our study demonstrated that cytokines IL-1β, IL-6, as well as severe CM, are significant predictors of lower general and rhinoconjunctivitis HRQoL in adolescents with AR. However, there are still some limitations of this study. Future research in larger targeted studies is necessary to thoroughly understand the mechanism underlying the up-regulation of cytokines in the nasal mucosa. Specifically, the effects of IL-1β, IL-6, IL-8, IL17-A, and IL-18 on nasal mucosa cells should be examined in vitro. Long-term, readily available biomarkers may make it easier to match patients with cutting-edge treatment modalities like anti-cytokine antibodies. Finding particular endotypes among clinically comparable phenotypes may lead to a more focused, customized treatment that is advantageous to the patient, particularly in terms of improved HRQoL. Due to the fact that this is the first time that inflammatory cytokines in nasal mucosa were correlated with HRQoL in an adolescent population with AR, positive results should only serve as a starting point for additional confirmation research.

## Figures and Tables

**Table 1:** Socio-demographic characteristics, severity of CM and comorbidities among groups of adolescents.

Variables	Allergic Rhinitis (*n* = 55)		Nonallergic Rhinitis (*n* = 34)		Control Group (*n* = 44)		Total (*n* = 133)		p ^†^
	*n*/ Mean	%/ SD	*n*/ Mean	%/ SD	*n*/ Mean	%/ SD	*n*/ Mean	%/ SD	
Gender									
Boys	23	41.8	14	50.0	21	47.7	61	45.9	0.720
Girls	32	58.2	17	50.0	23	52.3	72	54.1	
Age *	14.47 ± 1.69		14.26 ± 1.72		14.40 ± 1.66		14.39 ± 1.68		0.853
Age groups									
12 to 14 years	32	58.2	19	55.9	23	52.3	74	55.6	0.841
15 to 17 years	23	41.8	15	44.1	21	47.7	59	44.4	
Severity of CM (T4SS)									
No CM	0	0	0	0	44	100.0	44	100.0	**<0.001**
Mild CM	9	16.4	14	41.2	0	0	23	17.3	
Modern CM	22	40.0	12	35.3	0	0.0	34	25.6	
Severe CM	24	43.6	8	23.5	0	0.0	32	24.1	
Comorbidities (Yes)	46	83.6	24	70.6	0	0.0	70	52.6	**<0.001**
Sinusitis (Yes)	42	76.4	21	61.8	0	0.0	63	47.4	**<0.001**
Secretory otitis (Yes)	5	9.1	1	2.9	0	0.0	6	4.5	0.084
OSA (Yes)	3	5.5	1	2.9	0	0.0	4	3.0	0.287
Polyps (Yes)	0	0.0	2	5.9	0	0.0	2	1.5	0.052
Asthma (Yes)	8	14.5	0	0.0	0	0.0	8	6.0	**0.002**
AD (Yes)	18	32.7	7	20.6	0	0.0	25	18.8	**<0.001**
Conjunctivitis (Yes)	25	45.5	6	17.6	0	0.0	31	23.3	**<0.001**
Allergens the adolescents were sensitized to									
Mites (Yes)	34	61.8	0	0.0	0	0.0	34	61.8	**<0.001**
Birch tree pollen (Yes)	21	38.2	0	0.0	0	0.0	21	38.2	
Hazelnut pollen (Yes)	22	40.0	0	0.0	0	0.0	22	40.0	
Grass pollen (Yes)	29	52.7	0	0.0	0	0.0	29	52.7	
Cat’s tail grass pollen (Yes)	30	54.5	0	0.0	0	0.0	30	54.5	
Rye pollen (Yes)	21	38.2	0	0.0	0	0.0	21	38.2	
Ambrosia (Yes)	13	23.6	0	0.0	0	0.0	13	23.6	
Wild wormwood (Yes)	5	9.1	0	0.0	0	0.0	5	9.1	
Mold fungus *Aspergillus* *fumigatus* (Yes)	0	0.0	0	0.0	0	0.0	0	0.0	
*Alternaria* mold fungus (Yes)	2	3.6	0	0.0	0	0.0	2	3.6	
Brown cockroach (Yes)	5	9.1	0	0.0	0	0.0	5	9.1	
Cat hair (Yes)	3	5.5	0	0.0	0	0.0	3	5.5	
Dog hair (Yes)	6	10.9	0	0.0	0	0.0	6	10.9	
Type of AR									
SAR	35	63.6	0	0.0	0	0.0	35	63.6	**<0.001**
PAR	20	36.4	0	0.0	0	0.0	21	36.4	
Mono versus polysensitized subjects									
Mono-sensitized	21	38.2	0	0.0	0	0.0	21	38.2	**<0.001**
Poly-sensitized	34	61.8	0	0.0	0	0.0	34	61.8	

T4SS—Total 4 Symptom Score, OSA—Obstructive sleep apnea, CM—clinical manifestations, SAR—seasonal allergic rhinitis, PAR—perennial allergic rhinitis, AD—atopic dermatitis, * mean ± SD (standard deviation), ^†^ *p*—statistical significance measured by Mann-Whitney and χ^2^—chi square test. Significant values are bolded.

**Table 2:** Distribution of respondents according to the levels of the domain of quality of life measured by the EQ-5D-3L and AdolRQLQ questionnaires between groups of respondents.

Domains of Quality of Life Measured by the EQ-5D-3L and AdolRQLQ Questionnaires	Allergic Rhinitis (*n* = 55)		Nonallergic Rhinitis (*n* = 34)		Control Group (*n* = 44)		*p* *
	*n*/ Mean	%/ SD	*n*/ Mean	%/ SD	*n*/ Mean	%/ SD	
EQ-5D-3L							
Mobility							
No problems	42	76.4	31	91.2	44	100.0	
Moderate problems	13	23.6	3	8.8	0	0	**0.001**
Severe problems	0	0.0	0	0.0	0	0.0	
Self-care							
No problems	55	100.0	34	100.0	44	100.0	
Moderate problems	0	0.0	0	0.0	0	0.0	1.000
Severe problems	0	0.0	0	0.0	0	0.0	
Usual activities							
No problems	21	38.2	21	61.8	41	93.2	
Moderate problems	29	52.7	9	26.5	3	6.8	**<0.001**
Severe problems	5	9.1	4	11.8	0	0.0	
Pain/discomfort							
No problems	21	38.2	19	55.9	39	88.6	
Moderate problems	29	52.7	15	44.1	5	11.4	**<0.001**
Severe problems	5	9.1	0	0.0	0	0.0	
Anxiety/depression							
No problems	21	38.2	16	47.1	36	81.8	
Moderate problems	27	49.1	13	38.2	8	18.2	**<0.001**
Severe problems	7	12.7	5	14.7	0	0.0	
AdolRQLQ							
Practical problems	3.05 ± 1.15 ***		2.38 ± 1.45 #*		1.03 ± 0.14		**<0.001**
Symptoms unrelated to AR	2.65 ± 1.19 ***		2.23 ± 1.17 ***		1.01 ± 0.09		**<0.001**
Nasal symptoms	3.72 ± 1.41 ***		2.88 ± 1.46 ***		1.03 ± 0.16		**<0.001**
Ocular symptoms	2.38 ± 1.42 ***		1.59 ± 0.94 ##*		1.00 ± 0.00		**<0.001**
Activity limitation	2.24 ± 1.46 ***		1.45 ± 0.21		1.00 ± 0.00		**<0.001**
Emotional/mental problems	2.34 ± 1.22 ***		1.78 ± 0.99 #***		1.04 ± 0.21		**<0.001**

EQ-5D-3L—European Quality of Life 5 Dimensions 3 Level Version, AdolRQLQ—the Adolescent Rhinoconjunctivitis Quality of Life Questionnaire, *p*—statistical significance measured by χ^2^—chi square test or Kruskal–Wallis test, with pairwise post hoc testing between groups, * *p* < 0.05, *** *p* < 0.001 compared to the control group; # *p* < 0.05, ## *p* < 0.010 relative to allergic rhinitis group. Significant *p* values are bolded.

**Table 3:** Average concentrations of inflammatory cytokines of adolescents with AR divided into groups according to the severity of CM.

Inflammatory Cytokines (pg/mL)	Severity of Clinical Manifestations			*p*
	Mild CM (*n* = 9)	Modern CM (*n* = 22)	Severe CM (*n* = 24)	
	Mean ± SD	Mean ± SD	Mean ± SD	
IL-1β	40.90 ± 43.41 *	63.36 ± 94.99	72.77 ± 117.84	**0.012**
IFN-α2	5.58 ± 2.75	5.25 ± 0.85	5.22 ± 0.70	0.247
IFN-γ	15.10 ± 33.65	8.73 ± 14.20	5.89 ± 7.63	0.969
TNF-α	8.92 ± 20.35	5.25 ± 9.86	2.84 ± 2.50	0.903
MCP-1	27.68 ± 23.63	15.89 ± 13.40	18.63 ± 15.07	0.492
IL-6	14.68 ± 23.09 *	29.14 ± 9.40	48.99 ± 92.91	**0.028**
IL-8	1028.47 ± 407.36	1858.98 ± 1989.97	2103.39 ± 1971.34	0.317
IL-10	4.93 ± 7.89	2.71 ± 1.92	3.76 ± 6.51	0.288
IL-12p70	3.03 ± 3.09	1.93 ± 0.74	1.80 ± 0.53	0.323
IL-17A	0.80 ± 1.60 *	2.16 ± 0.70	4.50 ± 0.50	**0.021**
IL-18	42.61 ± 0.17 *	124.97 ± 141.47	142.46 ± 170.93	**0.018**
IL-23	13.59 ± 17.98	8.01 ± 6.09	8.69 ± 5.45	0.678
IL-33	23.25 ± 54.45	16.30 ± 22.45	9.68 ± 7.82	0.584

IL—interleukin, IFN—interferon, TNF-α—tumor necrosis factor alpha, MCP-1—Monocyte Chemotactic Protein-1, CM—clinical manifestations, SD—standard deviation, * *p* < 0.05 in relation to the severe form of allergic rhinitis, Kruskal–Wallis test, with pairwise post hoc testing between groups. Significant *p* values < 0.05 are bolded.

**Table 4:** Correlation of inflammatory cytokines from nasal mucosa of allergic rhinitis subjects with EQ-5D-3L and AdolRQLQ total scores.

Inflammatory Cytokines	Total EQ-5D-3L Score	EQ-VAS	Total AdolRQLQ Score
	r (*p*)	r (*p*)	r (*p*)
Il-1β	−0.116 (0.401)	−0.088 (0.525)	−0.008 (0.951)
IFN-α2	−0.207 (0.130)	−0.239 (0.079)	0.047 (0.734)
IFN-γ	0.056 (0.685)	0.111 (0.418)	−0.136 (0.323)
TNF-α	0.045 (0.742)	0.175 (0.200)	−0.127 (0.354)
MCP-1	0.005 (0.973)	−0.001 (0.994)	−0.045 (0.745)
IL-6	−0.114 (0.409)	−0.115 (0.405)	0.012 (0.932)
IL-8	−0.148 (0.281)	−0.093 (0.498)	−0.028 (0.837)
IL-10	0.083 (0.548)	0.155 (0.260)	−0.223 (0.102)
IL-12p70	0.240 (0.077)	0.228 (0.094)	0.299 **(0.027)**
IL-17A	−0.293 **(0.030)**	−0.247 (0.070)	0.150 (0.275)
IL-18	−0.184 (0.178)	−0.255 (0.060)	0.041 (0.767)
IL-23	−0.156 (0.256)	−0.137 (0.317)	0.002 (0.987)
IL-33	−0.151 (0.272)	−0.143 (0.298)	0.050 (0.717)

IL—interleukin, IFN—interferon, TNF-α—tumor necrosis factor alpha, MCP-1—Monocyte Chemotactic Protein-1, EQ-5D-3L—European Quality of Life 5 Dimensions 3 Level Version, EQ-VAS—European Quality of Life visual analog scale, AdolRQLQ—the Adolescent Rhinoconjunctivitis Quality of Life Questionnaire, r—Spearman coefficient of correlation, *p*—statistical significance. Significant *p* values are bolded.

**Table 5:** Regression analysis of the cytokine profile as a predictor of the quality of life (EQ-5D-3L and AdolRQLQ total scores) in models controlled for severity of clinical manifestations in adolescents with allergic rhinitis.

Variables	B	S.E.	β	*p*
EQ-5D-3L model				
IL-1β	0.301	0.112	0.400	**0.008**
IL-6	0.067	0.023	0.201	**0.043**
IL-8	0.112	0.000	0.047	0.807
IL-12p70	0.116	0.036	0.605	0.092
IL-17A	−0.267	0.066	−0.130	0.621
IL-18	0.281	0.000	0.117	0.335
Clinical manifestations	−0.160	0.040	0.136	**0.037**
Constant	1.014	0.118		**<0.001**
AdolRQLQ model				
IL-1β	0.421	0.026	0.389	**0.009**
IL-6	0.308	0.069	0.097	0.732
IL-8	0.000	0.000	−0.210	0.257
IL-12p70	−0.382	0.131	−0.522	0.246
IL-17A	0.528	0.241	0.431	0.433
IL-18	−0.001	0.001	−0.164	0.162
Clinical manifestations	0.877	0.148	0.230	**<0.001**
Constant	0.749	0.431		**0.005**

EQ-5D-3L—European Quality of Life 5 Dimensions 3 Level Version, AdolRQLQ—the Adolescent Rhinoconjunctivitis Quality of Life Questionnaire, B- unstandardized Coefficient, S.E—standard error, β—standardized coefficient, *p*—statistical significance. Significant *p* values are bolded.

## Data Availability

Data is unavailable due to privacy and ethical restrictions.
